# Book review: “Veterinary Parasitology, fifth edition” by Domenico Otranto and Richard Wall

**DOI:** 10.1186/s13071-025-06669-3

**Published:** 2025-01-27

**Authors:** Alicia Rojas

**Affiliations:** Laboratory of Helminthology, Faculty of Microbiology, University of Costa Rica, San José, Costa Rica

The fifth edition of the *Veterinary Parasitology* textbook written by Domenico Otranto and Richard Wall provides a fresh and practical view of the main parasitic agents of veterinary and zoonotic importance. The book is divided into two major sections to facilitate the flow of concepts. The first part focuses on the taxonomy, biology, and ecology of parasitic agents, with divisions by discipline: helminthology, protozoology, and entomology. Additionally, this section introduces general aspects of treatment strategies, resistance, epidemiology, and management approaches. The second part of the book provides a clear and comprehensive examination of the effects of parasites on various animal groups, including cattle, sheep and goats, horses, pigs, dogs and cats, poultry and game birds, ungulates, and even laboratory and exotic animals.

Several aspects of the book should be highlighted because of their usefulness and novelty:

1. The specifics of parasitic structures, apparatus, and body parts are explored in detail. This information is essential for understanding the parasite’s taxonomic classification, life cycle, infection pathways, and control strategies. Examples of these include the explanation of the structure and function of helminth and arthropod organs and systems.
2. This book provides a strong taxonomic basis with updated nomenclature on recently classified organisms such as *Dibothriocephalus latus* (formerly known as *Diphyllobothrium latum*) and *Eucoleus* spp. (which belonged to the *Capillaria* genus) to name a few. This demonstrates the thorough revision of literature dealing with synonyms, reclassifications, and new species descriptions.
3. Taxonomic keys for identification of gastrointestinal nematodes of ruminants, dipterans, lice, fleas, ticks, and mites are provided. These keys ease the identification of some parasitic groups that are usually difficult to classify. Importantly, this aspect is not common in other parasitology textbooks used for teaching purposes in the veterinary or medicine fields.
4. Illustrative guides and diagrams are provided for the identification of strongyles, acari, and ticks. These guides highlight key structures of each pathogen group to facilitate accurate classification. This approach addresses the challenges clinicians and researchers face when identifying these organisms. As a result, the illustrated guides simplify the process of comparing obtained specimens with established descriptions.
5. The new edition features eye-catching didactic life cycles that are simple, accurate, and informative. The diagrams shown in the book ease the understanding of this fundamental yet highly relevant aspect of parasitology. Gathering information on both well-established and newly described aspects of life cycles is usually a hard task. However, the illustrations in this new edition include detailed legends and incorporate information about intermediate, paratenic, and definitive hosts, as well as the anatomical and ecological niches explored by the parasites, directly within the images.
6. Comprehensive and explanatory tables outline the similarities and differences between species of some parasitic genera. For instance, several tables are provided for *Eimeria* spp. detailing the species, hosts, and anatomical sites of infection within this parasitic group. Another set of tables focus on the morphological characteristics of *Eimeria* spp. oocysts.
7. An innovative feature of the *Veterinary Parasitology* textbook is the inclusion of quick response (QR) codes linked to step-by-step video explanations of diagnostic protocols. These videos also feature researchers of different regions of the world, making the content more inclusive and accessible to diverse cultures. This not only simplifies the protocols, but also makes the procedures more familiar to anyone who wishes to perform them.
8. The chapters in part two, which focus on parasites by animal group, are concluded with a parasite checklist organized according to the host’s anatomical region. These tables serve as a quick reference for clinicians or researchers when faced with unknown specimens. They are particularly useful when basic aspects of the host group, organ, or system where the parasite was found are known, along with general knowledge of the parasitic group.
9. The glossary includes the etymology of the parasite’s scientific names, providing the Ancient Greek or Latin root, or in some cases, the slang from which the name is derived. This aspect not only helps in understanding the parasite’s ecological niche, host, or morphological characteristics, but also satisfies parasitologists’ need to learn more and more about these organisms.
10. There is a chapter devoted to parasitic agents of exotic animals. This new perspective addresses the needs of an expanding range of animal species with limited knowledge of the pathogens that may affect them.

The fifth edition of the *Veterinary Parasitology* textbook is an invaluable resource for veterinarians, biologists, microbiologists, physicians, and other professionals working with parasites derived from domesticated, pet, laboratory, or productive animals. The features outlined above underscore the novelty of this edition, demonstrating a commitment to enhancing previous versions by incorporating advanced technological resources and a greater number of illustrations and images.

